# Premonitory Signs of Severe Cerebral Disease

**Published:** 1852-02

**Authors:** 


*On the Premonitory Signs of Severe Cerebral Disease, and their Importance.*—By Dr. Devay, M. D.

1. *Premonitory signs, furnished by the intellectual faculties.*—Almost all authors of repute have mentioned, without always attaching much importance to them, the disturbances of intellect which precede attacks of severe cerebral disease. Insanity has its period of incubation, its premonitory symptoms; and frequently it is found that the first act of insanity, which caused alarm, has been preceded by several symptoms which had escaped observation, and sometimes the first phenomenon of the disease has been taken for its cause. The insane often combat their false ideas, before the disorder of their reason, and the internal contest which precedes the explosion of their madness, are perceived. The most general precursor of every severe affection of the brain is a state of *cerebral lassitude*, presenting much analogy to that state of intellectual torpor which follows severe or pestilential fevers. There is observed in the habitual gesture of the patients, in their attitudes and movements, a total absence of what may be called the consciousness of action. The brain seems to have lost its *balancing* power over the *ensemble* of the functions of the life of relation. These patients are often in a constant state of slight habitual vertigo, which they call *weakness of the head*, and which is frequently accompanied by debility in the limbs.

The *memory* is frequently impaired in the precursory period of cerebral affections. Thus, patients have forgotten the names of their friends, or of the most common things. In conversation, they have difficulty in finding proper words to express their meaning, and are obliged to make use of circumlocutions. More rarely, the memory becomes more powerful; it seems to take a new flight, and reproduces, to the great astonishment of the patient and his attendants, events which had seemed to be entirely forgotten. The curious and inexplicable fact of *reminiscence* corresponds to the exaltation of the special sensibility of certain senses. It is sometimes observed after a slight attack of apoplexy.

Next to the impairment of the memory, and also of the attention, which is fixed with difficulty, or not at all, on objects presented to the notice of the individual, the most striking change is in *volition*, which is diminished.—

The man who has hitherto been most firm, who has shown most tenacity in his views, who has pursued the plan of his life with great determination, becomes, in a measure, like the toy of a child ; those who are about him, even his inferiors, can command him. Human depravity has often taken advantage of this moral decadence for culpable ends ; and the man who has hitherto most rigorously and carefully managed his affairs, is all at once spoiled of his goods, either by extorted donations, or by burdensome expenses. The public see in these cases *bizarreries* of character ; the physiologist and physician see in them the first expression of a pathological condition. This weakening of the will, which, according to our observations, is chiefly connected with those cerebral lesions which lead to lunacy, or to paralysis of the insane, necessitates an alteration of the judgment. . . . . The will is the result of the other faculties ; and it is not because it is wanting in the idiot, or lunatic, that they are irresponsible ; but rather because they are ignorant of the rules which should direct it.

There is but a slight transition from this to *perversion of the moral faculties*—one of the most mysterious points in psychology.

The abrupt changes which may occur in a man's tastes, in his inclination, in his manner of living, in a word, in his social aspect, are worthy of attention. Modifications of this nature, when they do appear in a slow and progressive manner, do not arise from the action of moral influences, and can only arise from a change in the nervous system. Thus it has long been remarked, that unusual gaity in a habitually grave individual may denote the approach of an attack of apoplexy. It is the same with those who suddenly seek for noise and bustle, after having loved retirement and quietness for a great part of their life. We have known a man, aged 57, who, having up to that led a grave and even austere life, gave himself up to the pursuit of amusements unsuited to his age, and was, a few moments after, seized with sudden and complete apoplexy (*apoplexie foudroyante.*) A complete change in the turn of the ideas, when it is not the result of advanced age, when it manifests itself in a short period of time, and when it cannot be traced to the action of moral influences, is very suspicious. We have known a young physician who exhibited this phenomenon in a very marked manner, and who, a short time after, was seized with paralysis of the insane. When we knew him three years before, he was very free in his assertions, and inclined to exaggerate; but he had become discreet, and wary in his speech. His former condition, and the medium in which he had lived, showed sufficiently that this change could not be the effect of *progressive amendment;* we considered that there was some disease, and our opinion was ultimately confirmed.

It is conceivable, that the same psychological perturbation which changes the moral sentiments may likewise impair the sentiment of self-preservation; and hence that *suicidal melancholy* may mark the commencement of a severe affection of the brain. The disease is, moreover, very often conjoined with a lesion of the intellectual and affective faculties.

2. *Premonitory Signs furnished by the Sensorial Functions.*—Most of these are furnished by the sense of *vision.* We will merely mention dimness, the appearance of objects as if colored red, photophobia, &c., which may indicate threatening meningitis as well as cerebral hyperæmia; these symptoms bear an especial relation to acute diseases of the encephalon. These signs may exist several years before the explosion of the disease. Before attacks of apoplexy, impairment of vision sometimes exist in a high degree without being known to the patients, especially when, as is most commonly the case, it is not sufficient to prevent them from seeing those who are about them. The mistake is the more easy, as this symptom may be limited to one eye; the other compensating for the weakness of its fellow. Amblyopia is a frequent symptom; sometimes there is a complete blindness, as in the case of the Baron Hornestein, cited by Wepfer (*Anatomia Apoplecticorum,*) who became blind three weeks before a fatal attack of apoplexy.

A valuable sign, belonging in some degree to what may be called the expression of the eyes, consists in a want of parallelism in these organs; it is not squinting, nor is it the look of hallucination. It seems pretty well defined by the following expression: *The eyes are not in the axis of the reason.* There may be certain defects in this relation pointed out between a material object and a moral fact; but those persons who are accustomed to scrutinize the human look, and to see reflected in it the different passions will easily understand me.

The phenomena of exaltation of special sensibility, as a precursory sign of a severe encepalic lesion, is sometimes met with. It is in this case, as in other circumstances in which it is observed, one of the most mysterious problems for the physiologist. It is well known that heariug often becomes excessively acute before attacks of apoplexy. The patients, incommoded by the least noise, become irascible; they perceive distant sounds which are unheard by those who are with them. The fineness of hearing must be distinguished from the perception of strange and imaginary sounds, which is nothing but a sensorial hallucination.

The sense of *hearing* may present the same modifications as that of vision. Some persons are tormented with drumming in the ear, with continued or intermittent tinkling. Some believe that they hear the most strange noises. These hallucinations are by no means the constant precursors of an encephalic attack; they may be connected with simple perversions of the sensorial function.

*Premonitory Signs furnished by the Organs of Motion and Sensation.*—The alterations of the *muscular functions* present great variety, from the simple hesitation which we have already noticed, to paralysis which is complete, but which, on acconnt of its nature and its seat we shall denominate *irregular paralysis.* It is not uncommon to observe a state of general languor which makes the patients seek for rest—for the *far niente.* Van Swieten has remarked, in treating of apoplexy: *Primo oritur languor et amor quietis et otii.* At other times those who are about to be attacked with cerebral disease are much agitated, and expend a great amount of activity in their movements. Dr. Tessier has lately attended a lady, aged 60, who from the critical age, has been subject to attacks every month, at the period when she used to menstruate.—She loses consciousness; and after having recovered her senses, is paralysed on one side of the body, with great embarrassment of speech. These symptoms continue some days, and gradually leave her, to return at the fixed period. But some days before the new attack, this lady, though usually quiet and peaceable, exhibits much agitation; she cannot remain in her place, and those who are about her always know what this sign means. In this case we recognize an example of *periodic nervous apoplexy*.

Impairment of muscular motion is exhibited in various degrees. It is especially remarked in the lower limbs, which seem to bend under the weight of the body, and render the gait rather unsteady. The debility is the more striking if the person be young, and has no apparent cause for it. Portal was able to prognosticate an attack of apoplexy in a gentleman apparently in perfect health, from observing a slight fixedness in the left eye, and a slight weakness in the leg of the same side. *The dignitus semi-mortuus*, noticed by Dr. Marshall Hall, is one of those instances of *irregular paralysis*, of which it is so important to determine the true signification. Some time ago, we saw the following case:—A man, aged 54, one day called on us. In conversation, he jokingly noticed a sort of deadness which he felt in the little finger of the left hand, while the rest of the hand was able to perform its ordinary functions. We advised him to put himself under treatment: he neglected this advice, and some days after was seized with cerebral congestion, which left his faculties remarkably weakened. The *dignitus semi-mortuus* has shortly since been noticed in a valuable communication from Dr. Gillet de Grandmont.

Irregular paralysis, which seems to arise from exhaustion of the sources of the sensitive and motive powers, may appear under circumstances in which they do not constitute a symptom of such great importance. Such are those which sometimes follow hysterical convulsions, lead-colic, venereal abuses, &c. Here, these phenomena are connected with *transient* modifications of innervation. The suddenness of the attacks, their frequent isolation from other symptoms, their seat in parts distant from each other, while those lying between preserve the integrity of their movements, constitute the exceptional characters of those palsies which are connected with a latent alteration in the nervous centres. We must not lose sight of the difficulty of deglutition which some patients experience some time before being attacked; as well as the semi-paralysis of the vocal cords and tongue, giving rise to stammering or aphonia. The paralysis of the upper eyelids, which become œdematous, is also a sign of great value.

*Great sensibility* may be abolished, simply diminished or exaggerated. The first two forms almost always follow muscular paralysis; but they may exist alone. Sensibility may be exaggerated in two forms. The patients may present hyperæsthesia, or exquisite sensibility of the whole cutaneous surface; so that the least touch troubles them. This is an increased anormal sensibility—an exaggeration of the sense of touch, corresponding the exaltation of the sensorial faculties which we have already studied. Sensibility may also be exalted in the form of pain; and this merits our most careful attention. Violent pains, precursory of a severe cerebral lesion, have often been mistaken for neuralgia. The same is the case in treating cephalagia, supposed to be dependent on dyspepsia: and this error is more readily fallen into, as the stomach is often disordered. The diagnosis in these cases is sometimes difficult; but the duration and violence of the pain will lead to the suspicion, that there is something more than ordinary headache; and that, although the functions of the stomach are troubled at the same time, the headache is often too intense to be accounted for by the state of that organ. The patient cannot in general endure a warm room, nor the noise made by persons about him, nor even the fatigue of agreable conversation, without suffering an aggravation of his headache. The paroxysms are sometimes accompanied with vomiting, and sometimes with violent beating in the head. If with these symptoms we remark paleness of face, and weakness of pulse, and if active measures have been employed without benefit, we are led to suspect organic lesion.* Painful cramps are not unfrequent. Portal has seen patients who suffered severely from cramps in the legs before an attack of apoplexy.

Cutaneous sensibility presents other singular modes of perversion. A case is related of a man who, several months before being attacked with apoplexy, experienced from time to time an absolute loss of sensibility on five

*Abercrombie. Diseases of the Brain, p. 453.

or six isolated points of the skin of the thorax, each of about the size of a five-franc piece. Here the skin might be pinched without causing any pain; beyond, the sensibility was perfect. The partial abolitions of sensation were not constant. On some days there was not the least diminution of sensibility; then suddenly and simultaneously, it was annihilated in the isolated portions. Such unusual modifications of functions directly dependent on the brain, ought to furnish us with arguments in favor of the possibility of moral and instinctive perversions, and of tneir dependence, not on the corruption of the moral faculty itself, but on a latent pathological condition of the organ. Hence arises the doctrince of irresponsibility.

It is in the life of relation that indicatory signs are especially to be looked for. At the initial period of severe cerebral disease, organic life reveals few or no disturbances. The symptoms which may exist under this head only acquire value in connexion with those which are derived from the life of relation. The brain must be much affected to produce changes in the nutritive function.—Excepting sleep, which is one of the confines of animal and organic life, there is not in the latter any essential functional disturbanee. In the initial period, most patients have lost the power of sleep; or, if this function be performed, it is rather a fatiguing drowsiness than refreshing sleep. The digestive functions present no other special disorder than obstinate constipation, which is often difficult to be overcome by drastics. The eyelids sometimes become œdematous; and, in some subjects, attacks are preceded by small effusions of blood, even in the tissue of the conjunctiva. The secretions are but little altered. The urine is sometimes highly albuminous; but this is a subject for further researches.—*Rankins' Abstract.*

